# Genetic diversity and breed-informative SNPs identification in domestic pig populations using coding SNPs

**DOI:** 10.3389/fgene.2023.1229741

**Published:** 2023-11-16

**Authors:** Ichrak Hayah, Chouhra Talbi, Narjice Chafai, Isidore Houaga, Sara Botti, Bouabid Badaoui

**Affiliations:** ^1^ Laboratory of Biodiversity, Ecology, and Genome, Department of Biology, Faculty of Sciences, Mohammed V University in Rabat, Rabat, Morocco; ^2^ Plant and Microbial Biotechnologies, Biodiversity, and Environment (BioBio), Mohammed V University in Rabat, Rabat, Morocco; ^3^ Centre for Tropical Livestock Genetics and Health, The Roslin Institute, Royal (Dick) School of Veterinary Medicine, The University of Edinburgh, Edinburgh, United Kingdom; ^4^ The Roslin Institute, Royal (Dick) School of Veterinary Studies, University of Edinburgh, Edinburgh, United Kingdom; ^5^ PTP Science Park, Lodi, Italy; ^6^ African Sustainable Agriculture Research Institute (ASARI), Mohammed VI Polytechnic University (UM6P), Laâyoune, Morocco

**Keywords:** single nucleotide polymorphisms, informative markers, discriminant analysis of principal components, pig breeds, genetic diversity, functional enrichment analysis

## Abstract

**Background:** The use of breed-informative genetic markers, specifically coding Single Nucleotide Polymorphisms (SNPs), is crucial for breed traceability, authentication of meat and dairy products, and the preservation and improvement of pig breeds. By identifying breed informative markers, we aimed to gain insights into the genetic mechanisms that influence production traits, enabling informed decisions in animal management and promoting sustainable pig production to meet the growing demand for animal products.

**Methods:** Our dataset consists of 300 coding SNPs genotyped from three Italian commercial pig populations: Landrace, Yorkshire, and Duroc. Firstly, we analyzed the genetic diversity among the populations. Then, we applied a discriminant analysis of principal components to identify the most informative SNPs for discriminating between these populations. Lastly, we conducted a functional enrichment analysis to identify the most enriched pathways related to the genetic variation observed in the pig populations.

**Results:** The alpha diversity indexes revealed a high genetic diversity within the three breeds. The higher proportion of observed heterozygosity than expected revealed an excess of heterozygotes in the populations that was supported by negative values of the fixation index (F_IS_) and deviations from the Hardy-Weinberg equilibrium. The Euclidean distance, the pairwise F_ST_, and the pairwise Nei’s G_ST_ genetic distances revealed that Yorkshire and Landrace breeds are genetically the closest, with distance values of 2.242, 0.029, and 0.033, respectively. Conversely, Landrace and Duroc breeds showed the highest genetic divergence, with distance values of 2.815, 0.048, and 0.052, respectively. We identified 28 significant SNPs that are related to phenotypic traits and these SNPs were able to differentiate between the pig breeds with high accuracy. The Functional Enrichment Analysis of the informative SNPs highlighted biological functions related to DNA packaging, chromatin integrity, and the preparation of DNA into higher-order structures.

**Conclusion:** Our study sheds light on the genetic underpinnings of phenotypic variation among three Italian pig breeds, offering potential insights into the mechanisms driving breed differentiation. By prioritizing breed-specific coding SNPs, our approach enables a more focused analysis of specific genomic regions relevant to the research question compared to analyzing the entire genome.

## 1 Introduction

The domestic pig is an important livestock animal that is widely used for red meat, lard, and cured goods. It is a key player in the meat industry, particularly in Europe (OECD, 2022). Previous studies have suggested that the European domestic pig (*Sus scrofa* domesticus) is primarily descended from European wild boars (Giuffra et al., 2000). However, recent research has challenged this notion by identifying Asian mitochondrial DNA (mtDNA) haplotypes in European Yorkshire, Duroc, and Landrace pigs. This finding suggests that there may have been some interbreeding or genetic exchange between the two populations in the past (Giuffra et al., 2000; Larson et al., 2005). Throughout history, Italy has developed various breeds of pigs, each with unique characteristics and uses, such as Cinta Senese (Tuscany region), Nero Siciliano (Sicily region), and Mora Romagnola (Emilia-Romagna region) (Franci and Pugliese, 2007). The Yorkshire breed is one of the most commonly used commercial pig breeds and was introduced to Italy in the early 20th century due to its fast growth rate and high efficiency in converting feed into meat. The Landrace breed was introduced to Italy in the mid-20th century and has since been utilized in industrial pork production. The Duroc breed originated in the United States in the 19th century and has been exported to many countries, including Italy. This breed is often used in crossbreeding programs to produce hybrid pigs with desirable traits such as meat quality and growth rate (https://www.thepigsite.com/).

Both genetic and environmental factors have an impact on the phenotypic characteristics of commercial pig breeds, such as meat quality and disease resistance (Rosenvold and Andersen, 2003). Therefore, understanding the genetic diversity of these breeds is crucial for enhancing animal production, conserving animal genetic resources, and evaluating breed performance (Bovo et al., 2020; Dadousis et al., 2022). This research can help find breeds with better phenotypic traits and the ability to adapt to difficult conditions (Bovo et al., 2020). It can also support the sustainable growth of animal production in different settings and make it easier to reach evolutionary breeding goals rapidly (Notter, 1999).

The use of genome-wide panels of single nucleotide polymorphisms (SNPs) has transformed the study of pig breeds by allowing for the examination of complex relationships among them (Muñoz et al., 2019). However, processing such vast amounts of data can be challenging, leading to the need for a more efficient approach. One potential solution is to create less dense panels using a smaller set of markers specific to each breed based on a reduced number of SNPs. This approach would require less time and effort for analysis, thus making it more feasible. Breed-specific SNPs are frequently used in conservation biology to manage and protect livestock resources (Ozerov et al., 2013; Huisman, 2017), as well as for breed identification and authentication of meat and dairy products (Russo et al., 2007; Fontanesi et al., 2010).

The use of breed-informative SNPs has shown promising results in improving desired traits in pig breeding programs. A recent study on Italian Yorkshire pigs found that selecting SNPs associated with production traits, such as lean meat content, daily gain, and feed/gain ratio, can increase the frequency of desirable alleles over time, leading to faster improvement of these traits (Fontanesi et al., 2015). Genome-wide association studies (GWAS) have also become a popular way to find genetic variants linked to important production traits like meat and carcass quality, growth, and teat number in European pig breeds (Tang et al., 2019; Fabbri et al., 2020; Bovo et al., 2021). To identify breed-informative SNPs, various analytical tools, such as Random Forests, Principal Component Analysis, Regression, allele frequency differences, and Discriminant Analysis of Principal components, have been developed (Wilkinson et al., 2011; Schiavo et al., 2020; Hayah et al., 2021; Dadousis et al., 2022). These tools can help researchers identify key genetic markers and gain a deeper understanding of the genetic basis of production traits in pig breeds.

The aim of this study is to identify a breed-informative SNPs panel with high power to facilitate breed traceability and preservation efforts while also supporting breeding programs that prioritize desirable traits in these pig breeds. We anticipate that the identified SNPs will provide a useful tool for researchers and breeders alike, enabling them to make more informed decisions in animal management and breeding programs. By focusing on coding SNPs, we hope to identify genetic markers that are potentially functional, allowing for a better understanding of the underlying genetic mechanisms governing desirable production traits in commercial pig breeds. Ultimately, our research may contribute to the long-term sustainability of pig production, ensuring that we are able to meet the growing demand for animal products while preserving animal genetic diversity.

## 2 Materials and methods

### 2.1 Description of the dataset

#### 2.1.1 Source of data and SNP

The data utilized in this research is part of the MISAGEN project’s preexisting database (Botti et al., 2006; Biffani et al., 2011). This initiative gathered and archived a comprehensive dataset including pedigree information, clinical symptomatology, and health-related phenotypes from a commercial pig breeding population, which was sampled in Northern Italy. The initial dataset contained records from 2908 weaning piglets representing four distinct breeds: Yorkshire, Landrace, Duroc, and Pietrain. DNA extraction was carried out using nasal swabs as the source material. The subsequently extracted DNA was subjected to genotyping procedures employing the Illumina PorcineSNP60 BeadChip, designed to target a broad spectrum of over 60,000 Single Nucleotide Polymorphisms (SNPs) distributed across the pig genome.

#### 2.1.2 Quality control and SNP extraction

The genotyped data underwent rigorous quality control utilizing the quality control module within the GenABEL package of the R statistical software (Aulchenko et al., 2007). Specific criteria were set to exclude individual single nucleotide polymorphisms (SNPs):

▪ Exclusion of SNPs with a call rate less than 99% (i.e., SNPs not detected in at least 99% of all genotyped individuals).
▪ Removal of SNPs with a Minor Allele Frequency (MAF) in all individuals less than 0.05.
▪ Exclusion of individuals with a call rate less than 99% (i.e., individuals with more than 1% missing genotypes).
▪ Furthermore, individuals were excluded due to excessively high Identity By State (IBS) and sex discrepancies.

After applying these filters, a total of 14,967 SNPs (24.8% of the available 60,123 SNPs) and 77 individuals (0.063% of the total) were excluded from the analysis. In this study, a set of 300 coding SNP were chosen considering their physical proximity to genes linked to pig immunity. Plink software (Purcell et al., 2007) was used to extract those 300 coding SNPs from the three distinct pig populations: Yorkshire (YO), Landrace (LA), and Duroc (DU). Each breed was represented by 100 animals, resulting in a total of 300 animals analyzed in the study.

### 2.2 Data analysis

#### 2.2.1 Genetic diversity estimates

In this study, we used a range of genetic diversity metrics to analyze our dataset; all of the analyses were conducted in R software (R Core Team, 2020). All of the population genetics estimates reported in this work, including allele frequencies, expected (H_E_) and observed (H_O_) heterozygosity, the inbreeding coefficient (F_IS_), alpha (α) diversity indexes, exact tests for Hardy-Weinberg Equilibrium (HWE), under selection variants, and fixed alleles, were implemented using the “dartR” package (Gruber et al., 2022) and its dependencies from R statistical software. The genetic distances between breeds were implemented using the “dartR” package (Gruber et al., 2022) and its dependencies from R statistical software. The graphics were created using the “ggplot2” and “Graphics” packages (Hadley, 2016; R Core Team, 2020).

H_E_, H_O_, and F_IS_ were estimated according to Nei (Nei, 1987). Alpha diversity indexes for allelic richness (*q* = 0), Shannon information (*q* = 1), and heterozygosity (*q* = 2) were estimated according to Sherwin (Sherwin et al., 2017). The exact *p*-values for the HWE test were calculated using the method described by Wigginton (Wigginton et al., 2005), and the results were visualized using a ternary plot. We used the OutFlank method (Whitlock and Lotterhos, 2015) to find variants that were subject to selection pressures. This method involves figuring out the neutral fixation index (F_ST_) distribution from the actual data and then centering the distribution by fitting it to a chi-square model. Loci with a *p*-value of less than 0.05 were considered F_ST_ outliers and indicative of selection pressure. To estimate the pairwise F_ST_ values for genetic distances between pig breeds, we used Weir and Cockerham update of Wright’s approach (Wright, 1951; Weir and Cockerham, 1984), while we used Nei’s approach (Nei, 1987) to estimate the pairwise G_ST_ values for genetic distances between populations.

#### 2.2.2 Discriminant analysis of principal components (DAPC)

Our study implemented the Discriminant Analysis of Principal Components method with a three-fold purpose. Our first objective was to assess the discriminatory power of individual SNPs in distinguishing the three breed clusters. We aimed to optimize the separation of individuals into predefined groups using discriminant functions of principal components by maximizing between-group diversity and minimizing within-group diversity. Our second objective was to investigate the genetic structure of the population, considering the existing knowledge about the pig breeds and their genetic variation. Finally, our third objective was to determine the probability of animals joining a particular population based on their genetic background.

After identifying SNPs of significant importance, we utilized the Variant Effect Predictor (VEP) tool from the Ensembl database (McLaren et al., 2016) to compare them with the “Pig Reference (Sus_scrofa)” database. This comparison aimed to uncover the genes and biological pathways associated with these SNPs. Additionally, we conducted a search in the “NCBI database” using the SNP marker names as keywords to investigate their involvement in biological processes.

To analyze the population structure, we employed the “adegenet” package in the R software (Jombart, 2008) to perform Discriminant Analysis of Principal Components. Subsequently, we employed the “pca3d” package (Weiner, 2020) to visualize how the most significant SNPs segregated individuals into different clusters.

#### 2.2.3 Functional enrichment analysis (FEA) of the most discriminating SNPs between the pig breeds

To determine the crucial biological functions that differentiate our three pig breeds, we performed a Functional Enrichment Analysis on a gene list comprising the genes housing the most significant breed informative SNPs. We utilized the “gprofiler2” R package (Kolberg and Raudvere, 2021), which employs various databases such as the Gene Ontology (GO) database, Kyoto Encyclopedia of Genes and Genomes (KEGG), WikiPathways (WP), Human phenotype ontology (HP), and micro-RNA target (MIRNA) databases, among others. The gene list was automatically generated from our informative SNP set identifiers and served as the input for the “gost” function within the “gprofiler2” R package. This function conducts Functional Enrichment Analysis, utilizing the Gene Ontology database. Our analysis included a thorough statistical enrichment assessment using the hypergeometric test, and we applied multiple testing corrections to enhance result reliability. To minimize the potential for false positives, we established a user-defined threshold of 0.05.

## 3 Results

### 3.1 Genetic diversity within population and among pig breeds

#### 3.1.1 Genetic diversity within population

**FIGURE 1:**
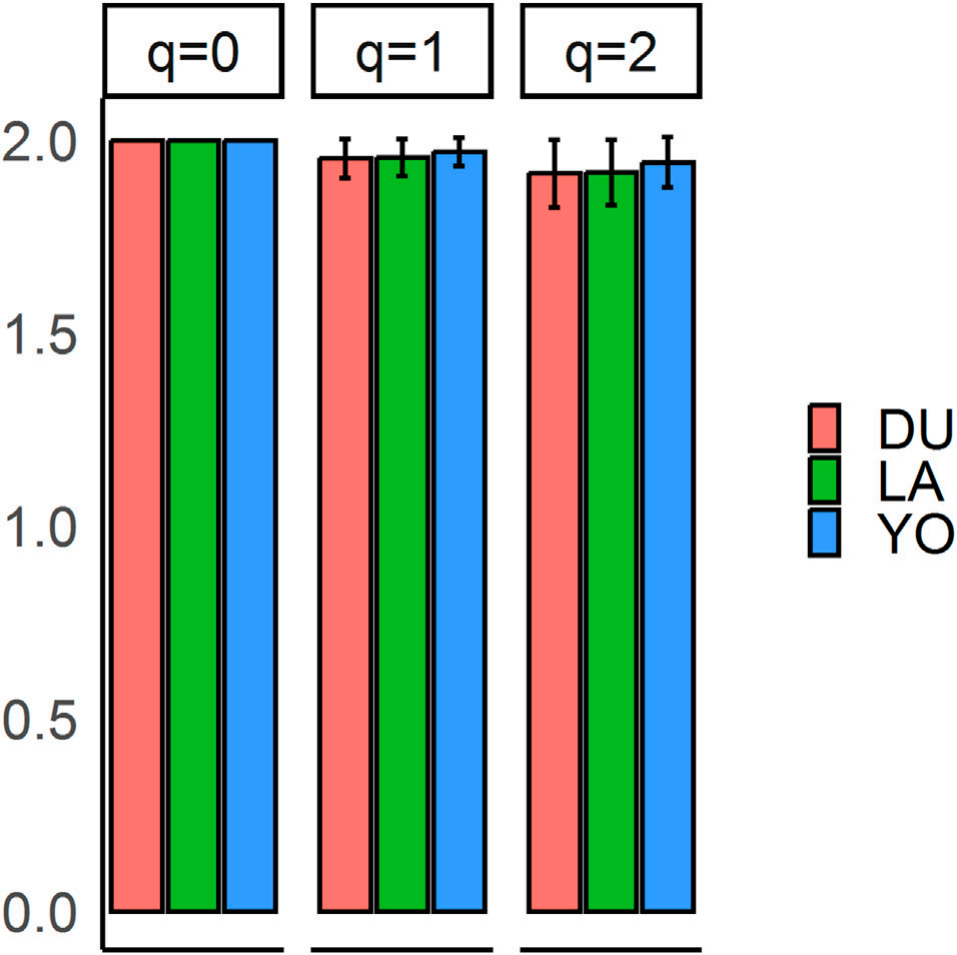
Alpha diversity q-profiles for the three populations. Allelic richness (*q* = 0), Shannon information (*q* = 1), and heterozygosity (*q* = 2).

**FIGURE 2:**
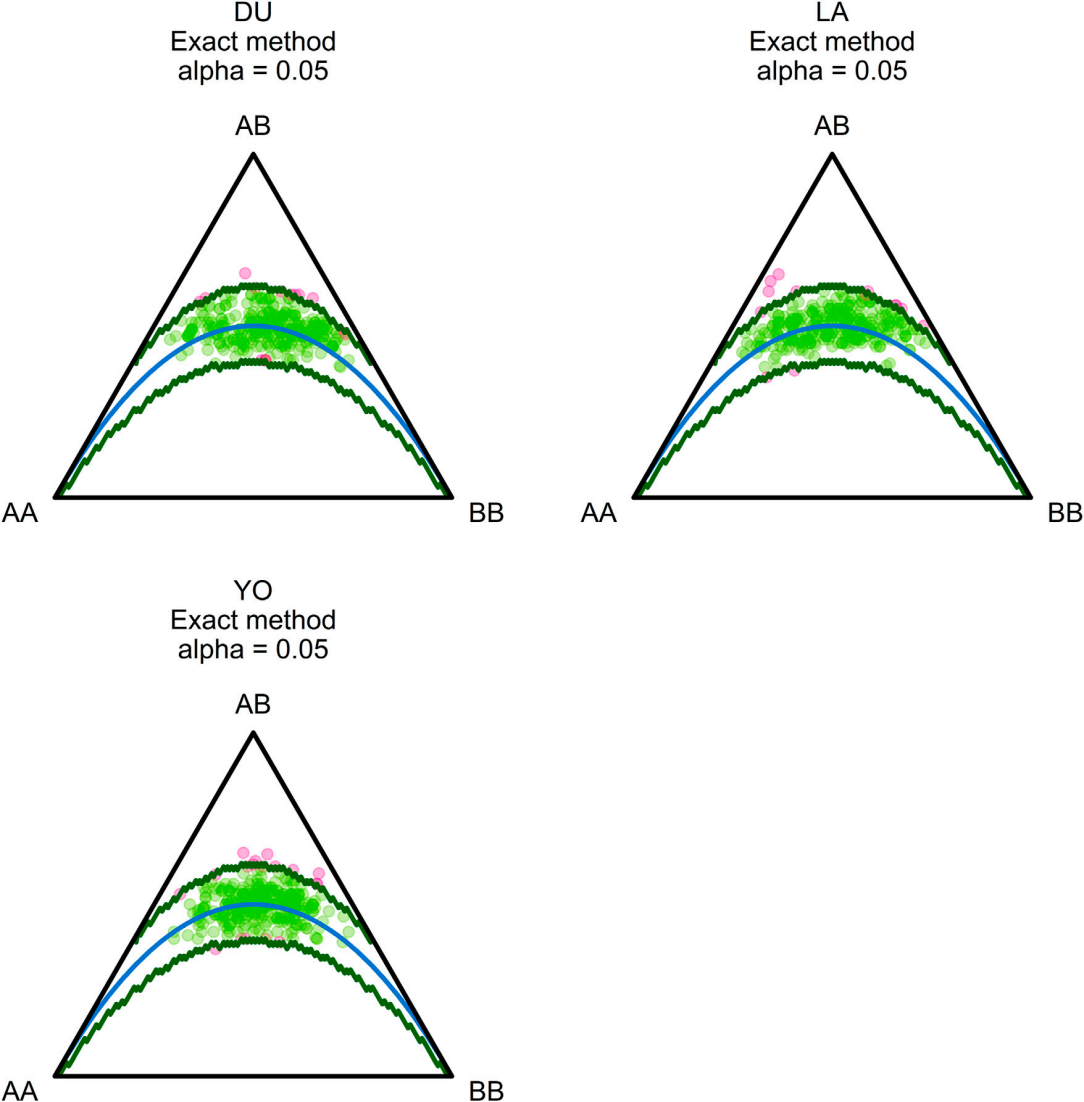
Ternary plots illustrating the patterns of Hardy-Weinberg (HW) proportions. Each vertex on the plot represents a different genotype: homozygous for the reference allele (AA), heterozygous (AB), and homozygous for the alternative allele (BB). The plots highlight loci that deviate significantly from Hardy-Weinberg equilibrium, and these loci are indicated in pink. The blue parabola on each plot represents Hardy-Weinberg equilibrium, while the area between the green lines represents the acceptance zone. The plots provide a visual representation of the distribution of the SNPs in relation to the Hardy-Weinberg equilibrium and allow for the identification of loci that may be under selection or experiencing other evolutionary forces.

The population sample shows a nearly equal proportion of the first and second alleles, with a slight preference towards the second allele (frequencies of 0.48 and 0.52, respectively). The observed proportion of heterozygotes in all three breeds is higher than expected, indicating a possible excess of heterozygotes. Our analysis of alpha diversity indexes reveals variability among different q-values, indicating a deviation from HWE. The average values of allelic richness, Shannon information, and heterozygosity are 2, 1.96, and 1.92, respectively (Figure 1). The negative value of the overall fixation index (F_IS_ = −0.03) supports this deviation from HWE. We conducted statistical tests to identify loci that deviate from HWE, and 46 SNPs showed statistically significant deviations (see Supplementary Table S1). These deviations are primarily concentrated at the vertex that represents heterozygotes (AB). The results of the chi-square test for selection pressure suggest that there is no evidence of selection acting on any of the loci, and the absence of fixed alleles in any of the three breeds supports this conclusion. The exact *p*-values of the test of HWE deviations are reflected in a ternary plot (Figure 2), with significant deviations indicated by pink dots. The blue parabola represents the expected genotype frequencies under HWE, and the space between the green lines indicates deviations that are not statistically significant.

#### 3.1.2 Genetic diversity/distance among the pig breeds

**FIGURE 3:**
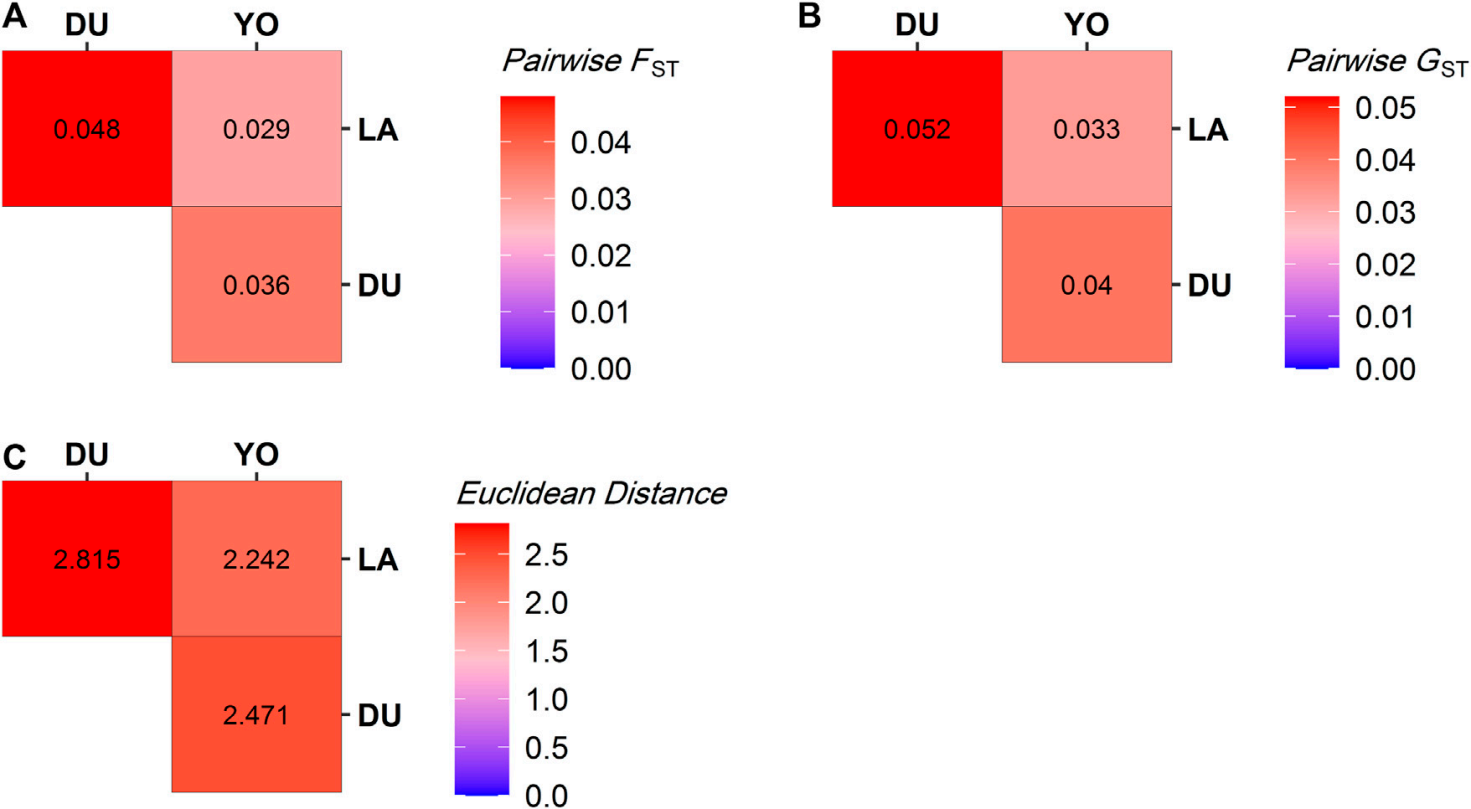
Distance measures between pig populations. **(A)** Pairwise F_ST_, **(B)** Pairwise G_ST_, and **(C)** Euclidean Distance. The warmer the color, the more the two breeds concerned are genetically distant.

We used Euclidean distance, pairwise F_ST_, and pairwise Nei’s G_ST_ to look at the genetic differences between the three groups of pigs. The heat maps in Figure 3 show the results. The heat maps indicate genetic divergence in red and genetic similarity in blue. Our analysis showed that the LA and DU breeds are the most genetically different from each other. Their estimated Euclidean distances are 2.815, their pairwise F_ST_ is 0.048, and Nei’s pairwise G_ST_ is 0.052, all of which show that they are very different genetically. Conversely, the YO and LA breeds were found to be the most genetically similar, with estimated Euclidean distances of 2.242, pairwise F_ST_ of 0.029, and Nei’s pairwise G_ST_ of 0.033, indicating a close genetic relationship between these two breeds.

### 3.2 Discriminant analysis of principal components (DAPC) to explore the pig populations structure

**FIGURE 4:**
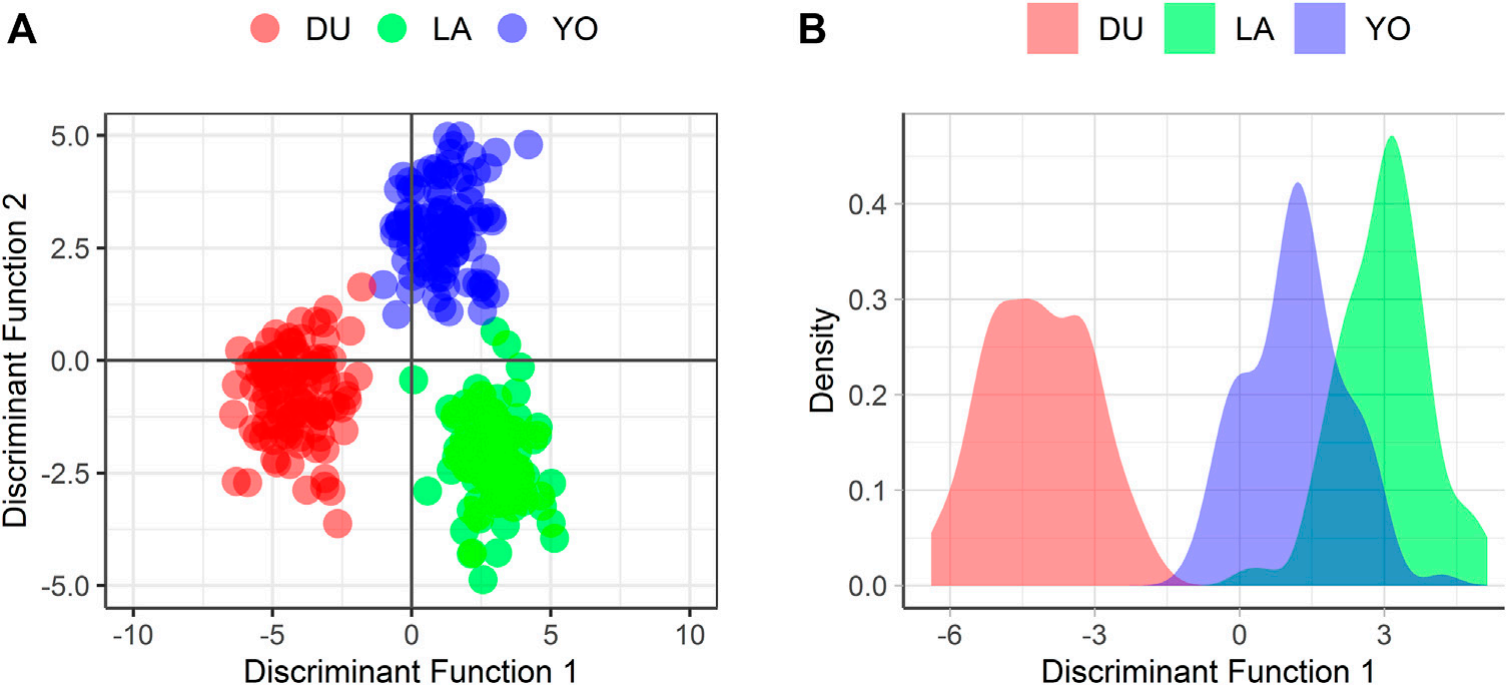
Visualization of the distribution of the 300 individuals according to the 300 SNPs **(A)** considering the first two discriminant functions, and **(B)** considering the first discriminant function only.

To further explore the population structure, we generated a DAPC plot based on the first and second Principal Components (PCs) (Figure 4A). We used the alpha-score optimization method (Jombart and Collins, 2015) to determine the necessary number of PCs. The clusters in the DAPC plot were defined by prior knowledge of population membership (K = 6). We retained 30 PCs, explaining 40% of the overall genetic variability, as input to the Discriminant Analysis.

**FIGURE 5:**
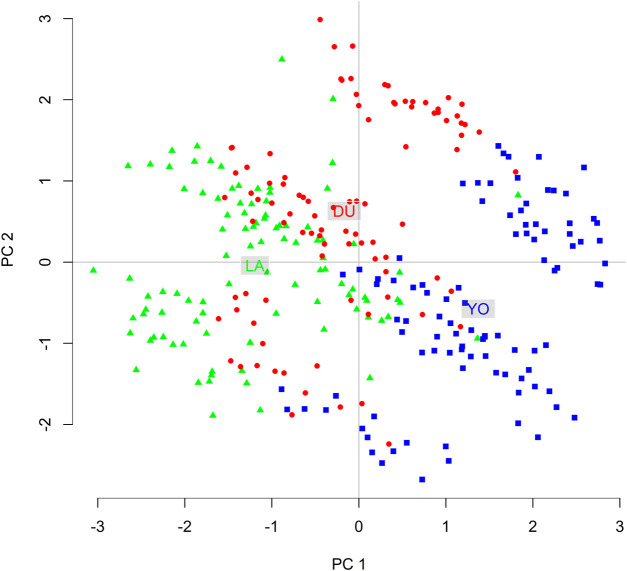
Two-Dimensional visualization of pig individuals distribution based on the 28 most informative SNPs using the first and second principal components.

**FIGURE 6:**
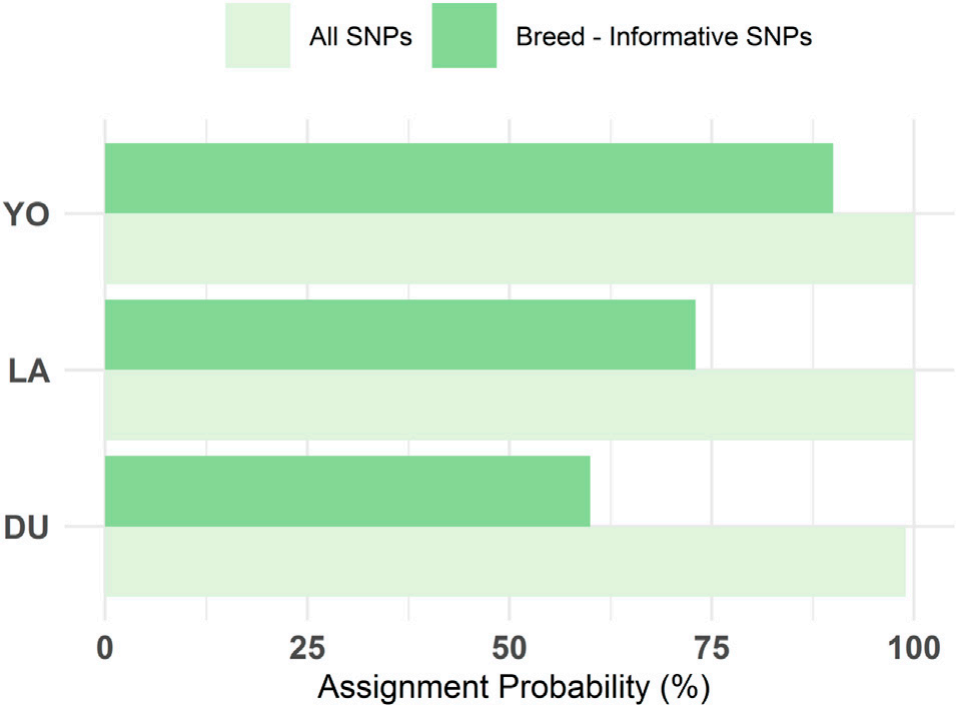
Comparison of the overall reassigning probability to actual breed estimated with DAPC using the initial 300 SNPs and the breed-informative selected 28 SNPs.

The DAPC plot showed clear clustering of individuals by breed, with the separation between breeds being more distinct in the first discriminant function (Figure 4B). The average assignment probability was 99% for DU and 100% for YO and LA breeds. We identified 28 SNPs that contributed most to breed differentiation based on a threshold of 0.01, and their names are listed in Supplementary Table S2. We performed a PCA on the 300-pig population using these 28 SNPs as variables, and the resulting plot showed clear clustering of individuals by breed (Figure 5). The reduced dataset’s overall assignment probability was 74%, with YO breeds having the highest assignment rates (90%), LA breeds coming in second (73%), and DU breeds coming in third (60%). The assignment rate using the whole dataset was higher compared to using only the most contributing SNPs. However, it is worth noting that the assignment rate achieved using the most informative SNPs remained notably high, standing at no less than 60% (Figure 6).

### 3.3 Functional enrichment analysis (FEA) of the most discriminating SNPs between the pig breeds

**FIGURE 7:**
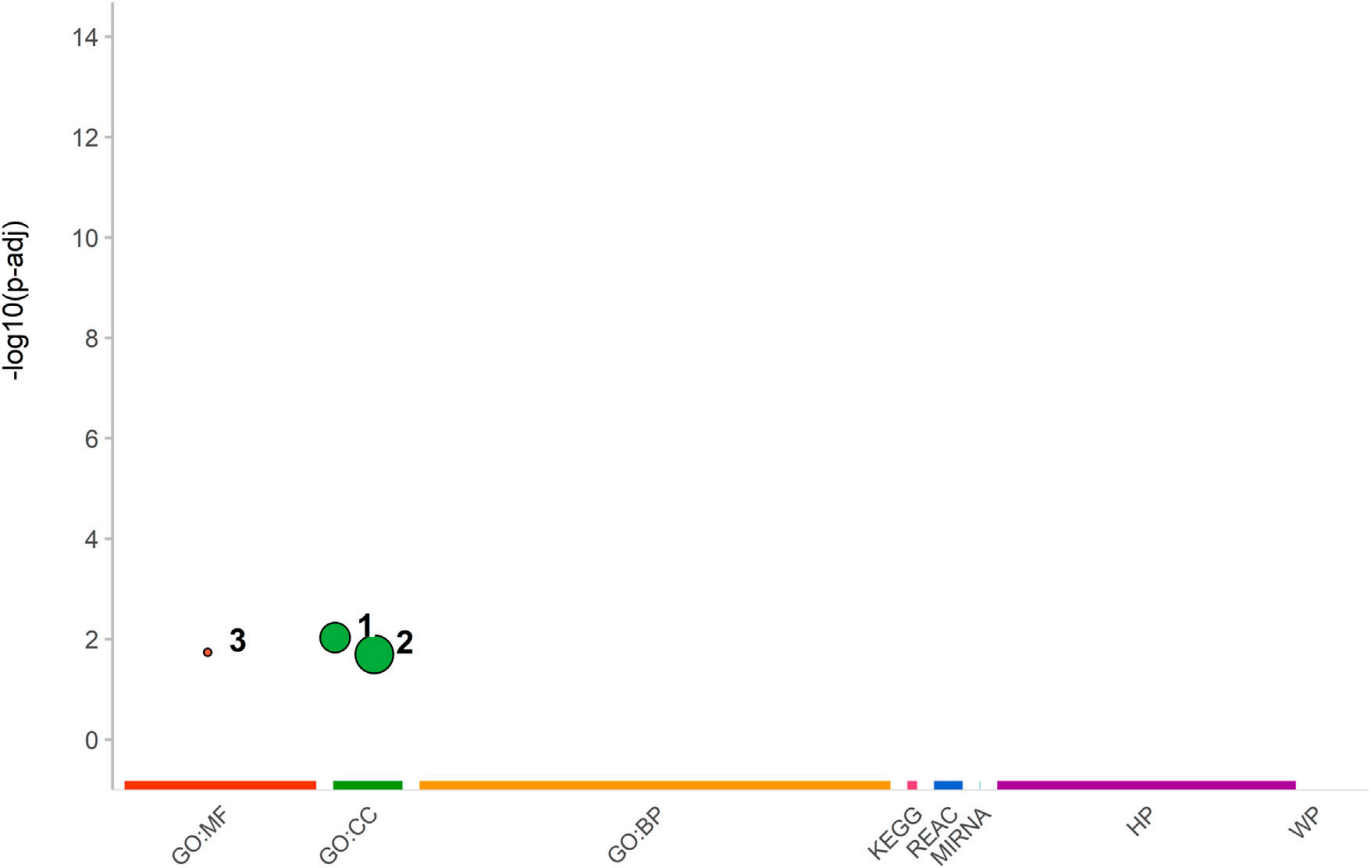
A graphical representation of the adjusted *p*-values in the negative log10 scale for enriched functions obtained from various databases, including Gene Ontology Molecular Functions (GO:MF), Gene Ontology Cellular Components (GO:CC), Gene Ontology Biological Processes (GO:BP), Kyoto Encyclopedia of Genes and Genomes (KEGG), Reactome Pathway (REAC), micro-RNA target (MIRNA), Human phenotype ontology (HP), and WikiPathways (WP). The enriched functions, namely, (1) nucleosome, (2) DNA packaging complex, and (3) structural component of chromatin, are plotted against their respective databases.

The functional Enrichment Analysis of the genes harboring the most breed informative SNPs revealed three important biological functions: (1) nucleosome, (2) DNA packaging complex, and (3) structural component of chromatin (Figure 7). These functions are crucial for regulating gene expression and maintaining DNA’s structural stability within the nucleus (Alberts et al., 2002). Nucleosomes are integral components of chromatin that organize and compact DNA into a condensed structure. The DNA packaging complex plays a crucial role in assembling and disassembling nucleosomes and regulating chromatin structure and function. The structural constituents of chromatin provide mechanical support to the chromatin fiber, maintaining its integrity. Table 1 presents the short names of these functions and their corresponding *p*-values, sorted in decreasing order of significance following hypergeometric testing and multiple testing adjustments.

**TABLE 1:** Top 3 significantly enriched functions according to their *p*-values.

ID	Source^a^	Term ID^b^	Term name^c^	Term size^d^	*p*-value
**1**	GO:CC	GO:0000786	Nucleosome	111	*9.3 e* ^*−*^ *^03^*
**2**	GO:CC	GO:0044815	DNA packaging complex	144	*2.0 e* ^*−*^ *^02^*
**3**	GO:MF	GO:0030527	Structural constituent of chromatin	82	*1.8 e* ^*−*^ *^02^*

^a^ The abbreviation of the data source for the term (Gene Ontology Molecular Functions (GO:MF), Gene Ontology Cellular Components (GO:CC)),

^b^ Unique term identifier,

^c^ The short name of the function,

^d^ Number of genes that are annotated to the term.

The *p*-values are below 0.01 which indicate that the observed enrichment is statistically significant.

## 4 Discussion

Through our study, we have uncovered the genetic diversity present in three commercially important pig breeds, namely, Landrace, Yorkshire, and Duroc. These findings hold significant implications for breeding programs and conservation initiatives focused on preserving the genetic diversity within pig populations.

During our investigation, we observed notable genetic variability in our coding variants across the three breeds. Additionally, the Hardy-Weinberg equilibrium test revealed deviations from the expected population equilibrium. We also noted variations in the diversity q-values and an overall negative F_IS_ value. The presence of an excess of heterozygosity in our dataset likely contributed to the observed HWE imbalance at 46 loci. It is noteworthy that our population does not appear to be subjected to selective pressure, and the deviations may be attributed to random mating among pig individuals, resulting in an isolate-breaking effect (Hamilton, 2021).

The identification of informative SNPs, particularly those located in coding regions, is crucial for developing cost-effective SNP panels to facilitate efficient genotyping and breeding selection. This approach can improve the accuracy and effectiveness of pig breeding programs, leading to the development of more robust and productive pig breeds (Fontanesi et al., 2015). Investigating coding SNPs is important for preventing genetic diseases caused by mutations in specific genes. By identifying these mutations and integrating them into breeding programs, the prevalence of these diseases in pig populations can be reduced, resulting in improved animal welfare and decreased economic losses for farmers (Mellencamp et al., 2008).

Previous research has identified informative SNPs for differentiating among various species, including cattle breeds (Cheong et al., 2013; Zwane et al., 2016; Bertolini et al., 2018) as well as wild boars and domestic pigs (Lorenzini et al., 2020). While previous studies have focused on identifying informative SNPs among commercial pig breeds (YO, DU, and LA) using non-coding SNPs (Schiavo et al., 2020; Hayah et al., 2021), our study aimed to identify informative SNPs using only coding variants.

In our study, we found 28 genetic markers (SNPs) that help distinguish the three pig breeds. Of these, six specific markers did not match what we expected based on the Hardy-Weinberg test.

The presence of these deviating SNPs highlights their importance as potential markers for distinguishing between the various pig breeds. However, it is essential to underscore that further comprehensive research and studies are imperative to validate and elucidate the precise roles and contributions of these SNPs in breed differentiation.

It is important to highlight that previous studies have already provided valuable insights into the implications of specific SNPs that we have identified in our research. For instance, a previous genome-wide association study (Große-Brinkhaus et al., 2015) demonstrated a significant association between the SNP *ALGA0039432* and boar taint as well as testes size parameters. This finding underscores the relevance of this particular SNP in relation to these specific traits.

Moreover, our analysis identified two SNPs, namely, *ALGA0060925* and *DRGA0005996*, as key contributors to breed differentiation. *ALGA0060925* is positioned downstream on chromosome 11 and is responsible for encoding a long non-coding RNA (lncRNA). In contrast, *DRGA0005996* is located on *SSC5* and corresponds to the *CPNE8* gene, which is responsible for producing the copine-8 protein. Copine-8 is a calcium-dependent phospholipid-binding molecule that plays a crucial role in calcium-mediated intracellular processes. It is worth noting that dysregulation of *CPNE8*, a member of the Copine family, has been associated with various diseases such as prion disease and gastric cancer in previous studies (Lloyd et al., 2013; Zhang et al., 2022). These findings suggest that *CPNE8* may have multifaceted roles beyond breed differentiation and warrants further investigation in relation to its potential involvement in disease pathways.

Furthermore, several other SNPs within our dataset have been previously associated with various phenotypic traits. For example, the intergenic variant *ASGA0077916* has demonstrated a significant correlation with the fatty acid composition of the Longissimus dorsi muscle (Sambache Tayupanta, 2016). Another SNP of interest, *ASGA0072056*, is located on *SSC16* within the *RETREG1* gene, responsible for encoding the reticulophagy regulator 1. Dysregulation of the *RETREG1* gene has been linked to the development of numerous diseases (Islam et al., 2018). In the context of viral diseases, other studies have highlighted the relationship between the absence of the *RETREG1* protein and heightened replication of Dengue and Zika viruses (Lennemann and Coyne, 2017). *ASGA0008283* is an intergenic variant on *SSC1*. *ASGA0072056* and *ASGA0008283* have been shown to be determinant factors in tracing the breeding farm of domesticated pigs (Kwon et al., 2017).

Lastly, *ALGA0078229* is situated on *SSC14* within the *RET* gene, which encodes the proto-oncogene tyrosine-protein kinase receptor *RET*. Dysregulation of *RET* has been implicated in the development of various tumor types (Zhao et al., 2023). Additionally, a previous study found a significant association between *ALGA0078229* and meat quality in German Landrace pigs (Ponsuksili et al., 2014).

Moreover, we conducted a comprehensive investigation to identify the biological processes associated with the SNPs that exhibited deviations from Hardy-Weinberg equilibrium. Notably, one genome-wide association study demonstrated a significant association between *ALGA0077162* and immune-relevant traits in the Landrace breed (Dauben et al., 2021). Additionally, *ASGA0050304* was identified as a quantitative trait locus strongly linked to intramuscular fat (IMF) in the gluteus medius (GM) and longissimus dorsi (LD) muscles of Duroc pigs (González Prendes, 2017).

Regarding the Functional Enrichment Analysis, our results have revealed three enriched functions that involve three important parts: the nucleosome, the DNA packaging complex, and the structural components of chromatin. These components play crucial roles in DNA packaging, organization, and gene expression, thereby ensuring the efficient functioning of critical nuclear processes such as transcription, replication, and DNA repair (Alberts et al., 2002). Nucleosomes were identified as the most significant function with the lowest *p*-value. Previous studies have demonstrated a correlation between increased circulating nucleosomes and inflammation as well as autoimmune diseases (Schwarzenbach et al., 2011; Pisetsky, 2012). Therefore, nucleosomes are believed to have the potential to initiate immune responses (Rönnefarth et al., 2006). Moreover, the activation of chromatin is vital for the immune response, with receptor engagement triggering reaction cascades that activate transcription factors and the chromatin template (Paz and Josefowicz, 2021). This synergistic activation of select genes is particularly evident in macrophages during inflammation, where they can rapidly express hundreds of genes (Paz and Josefowicz, 2021), thus highlighting the intricate relationship between chromatin dynamics and immune processes. Investigating these functions and their underlying molecular mechanisms could offer new insights into the regulation of gene expression associated with chromatin abnormalities.

In summary, our study highlights the effectiveness of DAPC in evaluating the genetic structure and admixture levels of pig breeds. The obvious breed-specific separation of individuals seen in the DAPC and PCA plots supports our findings that these three pig breeds have distinct genetic backgrounds. Despite using only coding variants, the SNPs selected by the DAPC approach were able to assign individuals to their respective breeds with a 74% probability of correct assignment. Although this may not match the assignment rate achieved with the full dataset, it is still a significant accomplishment and highlights the importance of carefully selecting impactful genetic markers for analysis. As a result, targeting coding regions associated with traits of interest provides a more straightforward analysis of genome-wide variants and yields more explicit results.

The SNPs discovered in this study have the potential to be used as markers for pig breed identification and conservation initiatives. Further research with larger sample sizes can provide a more comprehensive understanding of the genetic structure of these pig breeds and identify additional coding SNPs that contribute to breed differentiation. By conducting further investigations and experiments, we can gain a deeper understanding of the functional significance and underlying mechanisms of these identified SNPs.

## 5 Conclusion

This study highlights the significant genetic variation present in gene-coding regions among three Italian pig breeds. The Landrace and Duroc breeds were found to be highly divergent, while the Landrace and Yorkshire breeds exhibited closer genetic similarities. Notably, we identified 28 coding SNPs that were particularly informative in differentiating between these breeds, with enough genetic information to form distinct clusters of individuals. Investigating the signaling pathways and functional implications of these SNPs could provide valuable insights into the underlying genetic mechanisms that contribute to breed differentiation. While whole-genome analysis can determine genetic diversity, focusing on breed-specific coding SNPs can streamline the analysis by targeting specific regions relevant to the research question.

## Data Availability

The dataset analyzed for this study can be found in the European Variation Archive database: https://www.ebi.ac.uk/eva/?eva-study=PRJEB61260. Project: PRJEB61260. Analyses:562 ERZ17293001.

## References

[B1] Alberts B. Johnson A. Lewis J. Raff M. Roberts K. Walter P. (2002). “Chromosomal DNA and its packaging in the chromatin fiber,” in Molecular biology of the cell. 4th edition (New York, NY, USA: Garland Science). Available at: https://www.ncbi.nlm.nih.gov/books/NBK26834/ (Accessed May 25, 2023).

[B2] Aulchenko Y. S. Ripke S. Isaacs A. van Duijn C. M. (2007). GenABEL: an R library for genome-wide association analysis. Bioinformatics 23, 1294–1296. 10.1093/bioinformatics/btm108 17384015

[B3] Auwera G. van der O’Connor B. D. (2020). Genomics in the cloud: using docker, GATK, and WDL in terra. First edition. Sebastopol, CA, USA: O’Reilly Media.

[B4] Bertolini F. Galimberti G. Schiavo G. Mastrangelo S. Gerlando R. D. Strillacci M. G. (2018). Preselection statistics and Random Forest classification identify population informative single nucleotide polymorphisms in cosmopolitan and autochthonous cattle breeds. animal 12, 12–19. 10.1017/S1751731117001355 28643617

[B5] Biffani S. Botti S. Bishop S. C. Stella A. Giuffra E. (2011). Using SNP array data to test for host genetic and breed effects on Porcine Reproductive and Respiratory Syndrome Viremia. BMC Proc. 5, S28. 10.1186/1753-6561-5-S4-S28 PMC3108223 21645308

[B6] Botti S. Caprera A. Gaita L. Mondin P. Ossani N. Palermo S. (2006). “The misagen project: towards the genetic improvement of disease resistance of pig commercial populations,” in Proceedings of the 8th World Congress on Genetics Applied to Livestock Production, Belo Horizonte, Minas Gerais, Brazil, August, 2006.

[B7] Bovo S. Ballan M. Schiavo G. Ribani A. Tinarelli S. Utzeri V. J. (2021). Single-marker and haplotype-based genome-wide association studies for the number of teats in two heavy pig breeds. Anim. Genet. 52, 440–450. 10.1111/age.13095 34096632 PMC8362157

[B8] Bovo S. Ribani A. Muñoz M. Alves E. Araujo J. P. Bozzi R. (2020). Whole-genome sequencing of European autochthonous and commercial pig breeds allows the detection of signatures of selection for adaptation of genetic resources to different breeding and production systems. Genet. Sel. Evol. 52, 33. 10.1186/s12711-020-00553-7 32591011 PMC7318759

[B9] Cheong H. S. Kim L. H. Namgoong S. Shin H. D. (2013). Development of discrimination SNP markers for Hanwoo (Korean native cattle). Meat Sci. 94, 355–359. 10.1016/j.meatsci.2013.03.014 23567136

[B10] Dadousis C. Muñoz M. Óvilo C. Fabbri M. C. Araújo J. P. Bovo S. (2022). Admixture and breed traceability in European indigenous pig breeds and wild boar using genome-wide SNP data. Sci. Rep. 12, 7346. 10.1038/s41598-022-10698-8 35513520 PMC9072372

[B11] Dauben C. M. Pröll-Cornelissen M. J. Heuß E. M. Appel A. K. Henne H. Roth K. (2021). Genome-wide associations for immune traits in two maternal pig lines. BMC Genomics 22, 717. 10.1186/s12864-021-07997-1 34610786 PMC8491387

[B12] Fabbri M. C. Zappaterra M. Davoli R. Zambonelli P. (2020). Genome-wide association study identifies markers associated with carcass and meat quality traits in Italian Large White pigs. Anim. Genet. 51, 950–952. 10.1111/age.13013 33058170

[B13] Fontanesi L. Schiavo G. Scotti E. Galimberti G. Calò D. g. Samorè A. b. (2015). A retrospective analysis of allele frequency changes of major genes during 20 years of selection in the Italian Large White pig breed. J. Animal Breed. Genet. 132, 239–246. 10.1111/jbg.12127 25727360

[B14] Fontanesi L. Scotti E. Russo V. (2010). Analysis of SNPs in the KIT gene of cattle with different coat colour patterns and perspectives to use these markers for breed traceability and authentication of beef and dairy products. Italian J. Animal Sci. 9. 10.4081/ijas.2010.e42

[B15] Franci O. Pugliese C. (2007). Italian autochthonous pigs: progress report and research perspectives. Italian J. Animal Sci. 6, 663–671. 10.4081/ijas.2007.1s.663

[B16] Giuffra E. Kijas J. M. H. Amarger V. Carlborg Ö. Jeon J.-T. Andersson L. (2000). The origin of the domestic pig: independent domestication and subsequent introgression. Genetics 154, 1785–1791. 10.1093/genetics/154.4.1785 10747069 PMC1461048

[B17] González Prendes R. (2017). Genome-wide association analysis of meat quality and gene expression phenotypes in Duroc pigs. Available at: https://www.tdx.cat/bitstream/handle/10803/405245/rgp1de1.pdf?sequence=1 (Accessed September 29, 2022).

[B18] Große-Brinkhaus C. Storck L. C. Frieden L. Neuhoff C. Schellander K. Looft C. (2015). Genome-wide association analyses for boar taint components and testicular traits revealed regions having pleiotropic effects. BMC Genet. 16, 36. 10.1186/s12863-015-0194-z 25879925 PMC4429935

[B19] Gruber B. Georges A. Mijangos J. L. Pacioni C. Unmack P. J. Berry O. (2022). dartR: importing and analysing SNP and silicodart data generated by genome-wide restriction fragment analysis. Available at: https://CRAN.R-project.org/package=dartR (Accessed September 24, 2022).

[B20] Hadley W. (2016). ggplot2: elegant graphics for data analysis. Berlin, Germany: Springer-Verlag New York. Available at: https://ggplot2.tidyverse.org.

[B21] Hamilton M. B. (2021). Population genetics. Hoboken, New Jersey, United States: John Wiley & Sons.

[B22] Hayah I. Ababou M. Botti S. Badaoui B. (2021). Comparison of three statistical approaches for feature selection for fine-scale genetic population assignment in four pig breeds. Trop. Anim. Health Prod. 53, 395. 10.1007/s11250-021-02824-x 34245361

[B23] Huisman J. (2017). Pedigree reconstruction from SNP data: parentage assignment, sibship clustering and beyond. Mol. Ecol. Resour. 17, 1009–1024. 10.1111/1755-0998.12665 28271620 PMC6849609

[B24] Islam F. Gopalan V. Lam A. K.-Y. (2018). RETREG1 (FAM134B): a new player in human diseases: 15 years after the discovery in cancer. J. Cell Physiol. 233, 4479–4489. 10.1002/jcp.26384 29226326

[B25] Jombart T. (2008). adegenet: a R package for the multivariate analysis of genetic markers. Bioinformatics 24, 1403–1405. 10.1093/bioinformatics/btn129 18397895

[B26] Jombart T. Collins C. (2015). A tutorial for discriminant analysis of principal components (DAPC) using adegenet 2.0. 0. London: Imperial College London, MRC Centre for Outbreak Analysis and Modelling.

[B27] Kolberg L. Raudvere U. (2021). gprofiler2: interface to the “g:profiler” toolset. Available at: https://CRAN.R-project.org/package=gprofiler2 (Accessed November 25, 2022).

[B28] Kwon T. Yoon J. Heo J. Lee W. Kim H. (2017). Tracing the breeding farm of domesticated pig using feature selection (*Sus scrofa*). Asian-Australas J. Anim. Sci. 30, 1540–1549. 10.5713/ajas.17.0561 29073733 PMC5666188

[B29] Larson G. Dobney K. Albarella U. Fang M. Matisoo-Smith E. Robins J. (2005). Worldwide phylogeography of wild boar reveals multiple centers of pig domestication. Science 307, 1618–1621. 10.1126/science.1106927 15761152

[B30] Lennemann N. J. Coyne C. B. (2017). Dengue and Zika viruses subvert reticulophagy by NS2B3-mediated cleavage of FAM134B. Autophagy 13, 322–332. 10.1080/15548627.2016.1265192 28102736 PMC5324851

[B31] Lloyd S. E. Mead S. Collinge J. (2013). Genetics of prion diseases. Curr. Opin. Genet. Dev. 23, 345–351. 10.1016/j.gde.2013.02.012 23518043 PMC3705206

[B32] Lorenzini R. Fanelli R. Tancredi F. Siclari A. Garofalo L. (2020). Matching STR and SNP genotyping to discriminate between wild boar, domestic pigs and their recent hybrids for forensic purposes. Sci. Rep. 10, 3188. 10.1038/s41598-020-59644-6 32081854 PMC7035276

[B33] McLaren W. Gil L. Hunt S. E. Riat H. S. Ritchie G. R. S. Thormann A. (2016). The Ensembl variant effect predictor. Genome Biol. 17, 122. 10.1186/s13059-016-0974-4 27268795 PMC4893825

[B34] Mellencamp M. A. Galina-Pantoja L. Gladney C. D. Torremorell M. (2008). Improving pig health through genomics: a view from the industry. Dev. Biol. (Basel) 132, 35–41. 10.1159/000317142 18817284

[B35] Muñoz M. Bozzi R. García-Casco J. Núñez Y. Ribani A. Franci O. (2019). Genomic diversity, linkage disequilibrium and selection signatures in European local pig breeds assessed with a high density SNP chip. Sci. Rep. 9, 13546. 10.1038/s41598-019-49830-6 31537860 PMC6753209

[B36] Nei M. (1987). Molecular evolutionary genetics. New York, NY, USA: Columbia University Press. 10.7312/nei-92038

[B37] Notter D. R. (1999). The importance of genetic diversity in livestock populations of the future. J. Animal Sci. 77, 61–69. 10.2527/1999.77161x 10064028

[B38] OECD (2022). Meat consumption per capita: continued rise of poultry, pig meat and fall of beef. Paris: Organisation for Economic Co-operation and Development. Available at: https://www.oecd-ilibrary.org/fr/agriculture-and-food/meat-consumption-per-capita-continued-rise-of-poultry-pig-meat-and-fall-of-beef_066c3566-en (Accessed November 25, 2022).

[B39] Ozerov M. Vasemägi A. Wennevik V. Diaz-Fernandez R. Kent M. Gilbey J. (2013). Finding markers that make a difference: DNA pooling and SNP-arrays identify population informative markers for genetic stock identification. PLOS ONE 8, e82434. 10.1371/journal.pone.0082434 24358184 PMC3864958

[B40] Paz A. M. de Josefowicz S. Z. (2021). Signaling-to-chromatin pathways in the immune system. Immunol. Rev. 300, 37–53. 10.1111/imr.12955 33644906 PMC8548991

[B41] Pisetsky D. S. (2012). The origin and properties of extracellular DNA: from PAMP to DAMP. Clin. Immunol. 144, 32–40. 10.1016/j.clim.2012.04.006 22659033 PMC3724456

[B42] Ponsuksili S. Murani E. Trakooljul N. Schwerin M. Wimmers K. (2014). Discovery of candidate genes for muscle traits based on GWAS supported by eQTL-analysis. Int. J. Biol. Sci. 10, 327–337. 10.7150/ijbs.8134 24643240 PMC3957088

[B43] Purcell S. Neale B. Todd-Brown K. Thomas L. Ferreira M. Bender D. (2007). PLINK: a tool set for whole-genome association and population-based linkage analyses. Am. J. Hum. Genet. 81, 559–575. 10.1086/519795 17701901 PMC1950838

[B44] R Core Team (2020). R: a language and environment for statistical computing. Vienna, Austria: R Foundation for Statistical Computing. Available at: https://www.R-project.org/.

[B45] Rönnefarth V. M. Erbacher A. I. M. Lamkemeyer T. Madlung J. Nordheim A. Rammensee H.-G. (2006). TLR2/TLR4-independent neutrophil activation and recruitment upon endocytosis of nucleosomes reveals a new pathway of innate immunity in systemic lupus erythematosus. J. Immunol. 177, 7740–7749. 10.4049/jimmunol.177.11.7740 17114445

[B46] Rosenvold K. Andersen H. J. (2003). Factors of significance for pork quality—a review. Meat Sci. 64, 219–237. 10.1016/S0309-1740(02)00186-9 22063008

[B47] Russo V. Fontanesi L. Scotti E. Tazzoli M. Dall’Olio S. Davoli R. (2007). Analysis of melanocortin 1 receptor (*MC1R*) gene polymorphisms in some cattle breeds: their usefulness and application for breed traceability and authentication ofParmigiano Reggiano cheese. Italian J. Animal Sci. 6, 257–272. 10.4081/ijas.2007.257

[B48] Sambache Tayupanta J. E. (2016). Análisis genómico de la calidad de la carne y del metabolismo de los ácidos grasos en porcino. https://m.riunet.upv.es/bitstream/handle/10251/67871/SAMBACHE%20-%20AN%C3%81LISIS%20GEN%C3%93MICO%20DE%20LA%20CALIDAD%20DE%20LA%20CARNE%20Y%20DEL%20METABOLISMO%20DE%20LOS%20%C3%81CIDOS%20GRASOS%20EN%20.pdf?sequence=1&isAllowed=y.

[B49] Schiavo G. Bertolini F. Galimberti G. Bovo S. Dall’Olio S. Nanni Costa L. (2020). A machine learning approach for the identification of population-informative markers from high-throughput genotyping data: application to several pig breeds. Animal 14, 223–232. 10.1017/S1751731119002167 31603060

[B50] Schwarzenbach H. Hoon D. S. B. Pantel K. (2011). Cell-free nucleic acids as biomarkers in cancer patients. Nat. Rev. Cancer 11, 426–437. 10.1038/nrc3066 21562580

[B51] Sherwin W. B. Chao A. Jost L. Smouse P. E. (2017). Information theory broadens the spectrum of molecular ecology and evolution. Trends Ecol. Evol. 32, 948–963. 10.1016/j.tree.2017.09.012 29126564

[B52] Tang Z. Xu J. Yin L. Yin D. Zhu M. Yu M. (2019). Genome-wide association study reveals candidate genes for growth relevant traits in pigs. Front. Genet. 10, 302. 10.3389/fgene.2019.00302 31024621 PMC6459934

[B53] Weiner J. (2020). pca3d: three dimensional PCA plots. Available at: https://CRAN.R-project.org/package=pca3d (Accessed October 9, 2022).

[B54] Weir B. S. Cockerham C. C. (1984). Estimating F-statistics for the analysis of population structure. Evolution 38, 1358–1370. 10.1111/j.1558-5646.1984.tb05657.x 28563791

[B55] Whitlock M. C. Lotterhos K. E. (2015). Reliable detection of loci responsible for local adaptation: inference of a null model through trimming the distribution of F(ST). Am. Nat. 186 (Suppl. 1), S24–S36. 10.1086/682949 26656214

[B56] Wigginton J. E. Cutler D. J. Abecasis G. R. (2005). A note on exact tests of hardy-weinberg equilibrium. Am. J. Hum. Genet. 76, 887–893. 10.1086/429864 15789306 PMC1199378

[B57] Wilkinson S. Wiener P. Archibald A. L. Law A. Schnabel R. D. McKay S. D. (2011). Evaluation of approaches for identifying population informative markers from high density SNP Chips. BMC Genet. 12, 45. 10.1186/1471-2156-12-45 21569514 PMC3118130

[B58] Wright S. (1951). The genetical structure of populations. Ann. Eugen. 15, 323–354. 10.1111/j.1469-1809.1949.tb02451.x 24540312

[B59] Zhang P. Cao X. Guan M. Li D. Xiang H. Peng Q. (2022). CPNE8 promotes gastric cancer metastasis by modulating focal adhesion pathway and tumor microenvironment. Int. J. Biol. Sci. 18, 4932–4949. 10.7150/ijbs.76425 35982908 PMC9379401

[B60] Zhao L. Wang N. Zhang D. Jia Y. Kong F. (2023). A comprehensive overview of the relationship between RET gene and tumor occurrence. Front. Oncol. 13, 1090757. 10.3389/fonc.2023.1090757 36865807 PMC9971812

[B61] Zwane A. A. Maiwashe A. Makgahlela M. L. Choudhury A. Taylor J. F. Marle-Köster E. van (2016). Genome-wide identification of breed-informative single-nucleotide polymorphisms in three South African indigenous cattle breeds. South Afr. J. Animal Sci. 46, 302–312. 10.4314/sajas.v46i3.10

