# Supplementary material for: Genetic diversity and breed-informative SNPs identification in domestic pig populations using coding SNPs
Source: Front Genet. 2023 Nov 16;14:1229741. doi: 10.3389/fgene.2023.1229741 (PMC10687199; doi:10.3389/fgene.2023.1229741)
Supplement: Supplementary file 1 [file Table1.DOCX]

Supplementary Material

# Table S1. List of SNPs that do not apply to Hardy-Weinberg equilibrium sorted decreasingly according to their p-values

| **Population** | **Locus** | **Exact p-value** |
| --- | --- | --- |
| LA | MARC0026235 | 1,00E-04 |
| LA | M1GA0001107 | 1,00E-04 |
| LA | ALGA0005737 | 8,00E-04 |
| LA | MARC0031395 | 0.0014 |
| DU | ISU10000003 | 0.0043 |
| YO | H3GA0030144 | 0.0045 |
| YO | MARC0112715 | 0.0047 |
| DU | ALGA0062343 | 0.0081 |
| LA | ALGA0039432 | 0.0097 |
| LA | ASGA0099719 | 0.013 |
| YO | MARC0027929 | 0.0163 |
| DU | ALGA0020050 | 0.02 |
| YO | ASGA0096207 | 0.021 |
| LA | ALGA0060925 | 0.0213 |
| LA | ALGA0081924 | 0.0225 |
| YO | M1GA0013156 | 0.0225 |
| LA | ALGA0105697 | 0.023 |
| LA | ALGA0111072 | 0.023 |
| LA | ALGA0008966 | 0.0243 |
| YO | ALGA0031122 | 0.0243 |
| YO | ALGA0031124 | 0.0243 |
| LA | ASGA0099925 | 0.0243 |
| YO | DRGA0011358 | 0.0263 |
| YO | DIAS0001046 | 0.0279 |
| DU | ASGA0053297 | 0.0307 |
| DU | INRA0015922 | 0.0329 |
| DU | ALGA0077162 | 0.0351 |
| YO | ASGA0084555 | 0.0354 |
| DU | ALGA0055156 | 0.0375 |
| YO | ASGA0063064 | 0.039 |
| DU | ASGA0050304 | 0.0405 |
| LA | ALGA0115762 | 0.0408 |
| DU | H3GA0012543 | 0.0412 |
| LA | ALGA0076523 | 0.0418 |
| YO | H3GA0042395 | 0.0423 |
| DU | DBWU0000709 | 0.0428 |
| YO | H3GA0056333 | 0.0439 |
| YO | ALGA0050070 | 0.0444 |
| DU | MARC0003111 | 0.0447 |
| DU | ALGA0076523 | 0.0471 |
| YO | ALGA0114080 | 0.0471 |
| DU | ALGA0089982 | 0.0474 |
| DU | ASGA0072828 | 0.0474 |
| YO | M1GA0004410 | 0.0474 |
| YO | M1GA0024771 | 0.0474 |
| DU | MARC0105115 | 0.0474 |
