# Supplementary material for: Genetic diversity and breed-informative SNPs identification in domestic pig populations using coding SNPs
Source: Front Genet. 2023 Nov 16;14:1229741. doi: 10.3389/fgene.2023.1229741 (PMC10687199; doi:10.3389/fgene.2023.1229741)
Supplement: Supplementary file 2 [file Table2.DOCX]

Supplementary Material

# Table S2. List of the most contributing SNPs to DAPC's Principal components

| **SNP Name** | **Contribution value** |
| --- | --- |
| MARC0053644 | 0.022 |
| ASGA0042644 | 0.022 |
| DRGA0005996 | 0.021 |
| ALGA0060925 | 0.021 |
| MARC0008262 | 0.02 |
| ALGA0078229 | 0.02 |
| ALGA0051902 | 0.019 |
| MARC0065987 | 0.019 |
| ALGA0109325 | 0.018 |
| ALGA0039432 | 0.018 |
| ASGA0072056 | 0.017 |
| ASGA0077181 | 0.016 |
| ASGA0077916 | 0.016 |
| ALGA0005737 | 0.015 |
| ASGA0010040 | 0.015 |
| ALGA0050654 | 0.015 |
| MARC0026235 | 0.014 |
| M1GA0001107 | 0.013 |
| ASGA0008283 | 0.013 |
| ALGA0016525 | 0.013 |
| ASGA0093046 | 0.012 |
| MARC0031395 | 0.012 |
| H3GA0016158 | 0.011 |
| ALGA0048346 | 0.011 |
| ASGA0060845 | 0.011 |
| MARC0109216 | 0.01 |
| ALGA0055075 | 0.01 |
| ASGA0054861 | 0.01 |
